# Proteomic profiling revealed unique disease biology associated with 1q abnormalities in multiple myeloma

**DOI:** 10.1038/s41698-026-01364-7

**Published:** 2026-04-14

**Authors:** Kiran K. Mangalaparthi, Joel-Sean Hsu, J. Erin Wiedmeier-Nutor, Partho Sen, Julie Staub, Firdous A. Bhat, Caleb K. Stein, Greg J. Ahmann, Shaji K. Kumar, S. Vincent Rajkumar, P. Leif Bergsagel, Rafael Fonseca, Esteban Braggio, Richard K. Kandasamy, Akhilesh Pandey

**Affiliations:** 1 Department of Laboratory Medicine and Pathology, Mayo Clinic, Rochester, MN USA; 2 Department of Biochemistry and Molecular Biology, Mayo Clinic, Rochester, MN USA; 3 Division of Hematology and Medical Oncology, Mayo Clinic, Phoenix, AZ USA; 4 Division of Hematology, Mayo Clinic, Rochester, MN USA; 5 Department of Immunology, Mayo Clinic, Rochester, MN USA; 6 Department of Quantitative Health Sciences, Mayo Clinic, Rochester, MN USA; 7 Manipal Academy of Higher Education, Manipal, India; 8 Center for Individualized Medicine, Mayo Clinic, Rochester, MN USA

**Keywords:** Biomarkers, Cancer, Computational biology and bioinformatics, Genetics, Oncology

## Abstract

Gain or amplification of chromosome 1q (+1q) is a common genomic alteration occurring in the plasma cells in nearly 40% of multiple myeloma patients. Although it is associated with inferior outcomes and is more common in the relapsed or refractory stages, the impact of +1q at the proteomic level remains unclear. Here, we studied enriched CD138+ plasma cells in newly diagnosed multiple myeloma to uncover molecular alterations associated with +1q. Differential expression analysis revealed significantly increased expression of over 100 proteins encoded by the 1q region, indicating a potential gene dosage effect. Pathway enrichment analysis identified enrichment of cell cycle proteins such as CDK1, MCM complex, CHEK2, PSME3 and NEK7 in cases with +1q gain. Further, protein-protein interaction network analysis showed enrichment of MYC transcriptional targets in +1q cases, including increased expression of TIPRL that is encoded on 1q24. In agreement with these findings, increased *TIPRL* transcript expression was correlated with +1q across different cytogenetic subgroups in the CoMMpass dataset. Further, high *TIPRL* expression was associated with poor prognosis in patients from the hyperdiploidy subgroup. Overall, this study highlights the role of proteomics in understanding molecular events associated with chromosomal alterations in MM and identifying potential targets for further functional analysis.

## Introduction

Multiple myeloma (MM) is a plasma cell neoplasm characterized by distinct cytogenetic abnormalities that define disease manifestations and outcomes. Primary abnormalities in MM include the hyperdiploid karyotype, characterized by extra copies of odd chromosomes 3, 5, 9, 11, 15, 19, and 21, and chromosomal translocations involving the immunoglobulin heavy chain (IgH) locus with multiple chromosomal partners^1^. While these primary events define heterogenous disease groups, secondary abnormalities frequently emerge during disease progression that include amplification or gain or amplification of chromosome 1q (+1q), deletion of 1p, deletion or mutation of 17p (*TP53*), monosomy 13, and translocations of MYC^1^. Both primary and secondary cytogenetic alterations significantly influence risk stratification, therapeutic response and clinical outcomes.

+1q is one of the most common secondary abnormalities in MM, occurring in ~30–40% of newly diagnosed patients with an even higher incidence observed in relapse/refractory MM^2–4^. Several studies have shown that +1q is associated with unfavorable patient outcomes, particularly when the abnormality includes 4 copies or more (i.e., amplification)^5–7^. Patients with +1q experience shorter progression-free survival and overall survival compared to those with normal 1q status when treated with triplet regimens of lenalidomide, bortezomib and dexamethasone^8^. Further, the presence of +1q along with other cytogenetic alterations such as t(4;14), t(14;16) and del(17p) has an adverse effect on survival highlighting the severity of this abnormality in the disease progression^9^. Despite its well-established clinical relevance, the global molecular alterations associated with +1q remain poorly characterized, underscoring a major gap in our understanding of this high-risk subgroup in multiple myeloma.

Several genes located on chromosome 1q, including *CKS1B*, *BCL9*, *MCL1*, *UBE2Q1*, *IL6R*, *ADAR1*, *PSMD4*, *MUC1* and *ILF2*, are considered oncogenic drivers owing to their ability to promote cell growth or drug resistance^10–16^. Notably, previous studies that primarily focused on transcript-level changes have identified gene signatures associated with high-risk disease, with several of these genes belonging to the 1q region^17,18^. However, the efforts to characterize the +1q in MM have largely been limited to transcriptomic analysis. Importantly, multiple studies across different cancers have reported only moderate correlations between RNA to protein abundance^19^, reflecting the role of post-transcriptional, translational and post-translational regulatory mechanisms. As protein levels often directly correlate with the disease phenotype, there is a need to investigate protein-level alterations associated with +1q in MM. Despite recent multi-omic studies investigating the impact of +1q on MM patients^13,20^, no large-scale proteomic characterization of +1q MM has yet been reported. In this study, we analyzed enriched CD138+ cells from 51 MM patients with known status of +1q alteration. Our goal was to investigate the proteomic and transcriptomic profiles of +1q MM and to compare them to cases with normal copy numbers of 1q to obtain deeper insights into the molecular landscape of +1q MM.

## Results

### Proteomic analysis of CD138+ myeloma cells

**Fig. 1:**
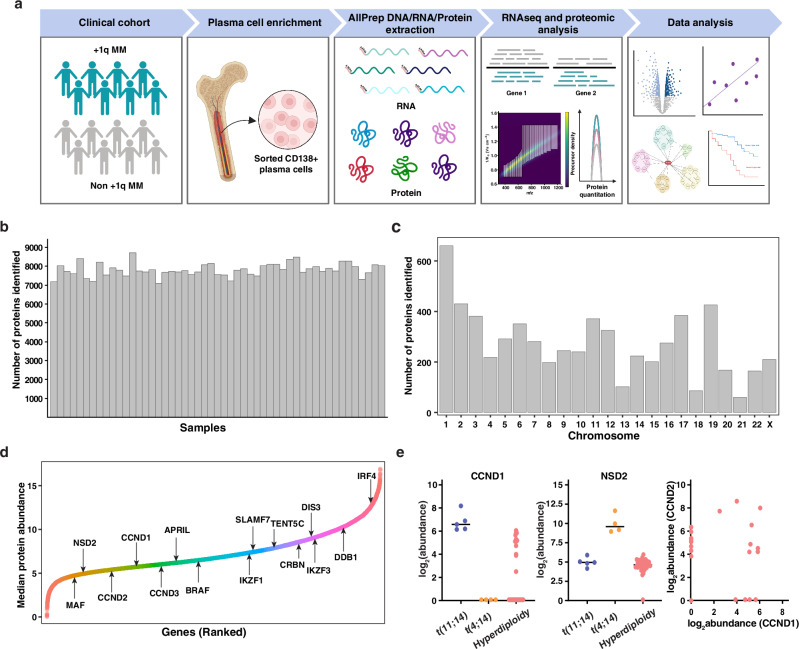
Summary of the proteomic analysis of CD138+ plasma cells from MM cases. **a** Experimental design for performing the transcriptomic and label-free quantitative proteomic analysis of enriched CD138+ plasma cells from MM cases with and without +1q. **b** Summary of the number of protein identifications across all the samples. **c** Number of proteins quantified across all the chromosomes. **d** Ranked distribution of the protein abundance across the samples indicating higher expression of plasma cell markers such as IRF4, IKZF1 and IKZF3. **e** Expression of CCND1 (left) and NSD2 (middle) across t(11;14), t(4;14) and hyperdiploidy MM. Scatter plot of abundance of CCND1 and CCND2 (right) across the hyperdiploidy samples.

The enriched CD138+ cells from 51 MM cases used in this study include 21 cases with +1q (3 copies) and 30 cases without +1q. The clinical details for these cases are summarized in Supplementary Data 1. An unbiased proteomic analysis of CD138+ cells was performed using a mass spectrometry-based diaPASEF workflow (Fig. 1A). This analysis led to the identification of 10,110 proteins, with a median of 7762 proteins identified per sample (Fig. 1B) (Supplementary Data 2). Further, a similar number of proteins were identified across cases with or without +1q (~7700 vs ~7800 proteins) and 7188 proteins quantified in at least 60% of cases in each group were used for downstream analyses. This in-depth analysis enabled the characterization of protein expression across all the chromosomes with over 600 proteins mapped to chromosome 1 (Fig. 1C). Several key plasma cell-enriched proteins such as transcription factors IKZF1, IKZF3 and IRF4 were identified with high protein expression across the samples (Fig. 1D). To establish the robustness of the global proteomic analysis, protein-level expression was first assessed for genes that are dysregulated as a consequence of primary genomic alterations. As expected, a higher expression of CCND1 was observed in cases with t(11;14), and similarly, NSD2 expression was higher in t(4;14) cases^21,22^ (Fig. 1E). CCND1 and CCND2 expression were mutually exclusive across t(4;14) and t(11;14) cases and also among hyperdiploidy samples, except for a subset of cases displaying a D1 + D2 phenotype as reported previously^21^. Overall, these findings indicate that proteomic analysis captured the established findings that are central to plasma cell biology in MM.

### Integrated RNA and protein expression analysis revealed positive correlation of genes encoded by chromosome 1q region

**Fig. 2:**
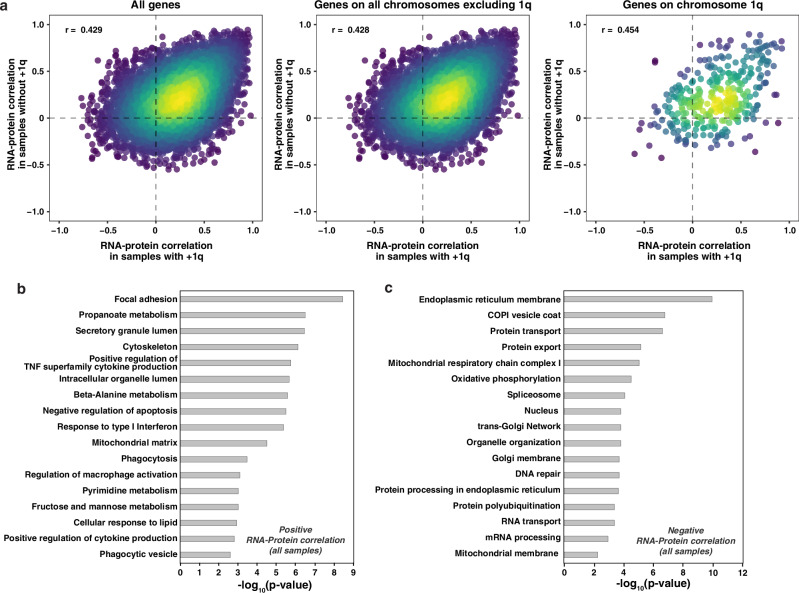
Integrative analysis of RNA and protein abundance across the cases with and without +1q. **a** Scatter plot of the Pearson correlation of RNA and protein levels of all the genes between cases with and without +1q (left), genes excluding those encoded by 1q chromosomal region (middle) and only genes encoded by 1q region (right). **b** Pathways enriched among the genes with positive correlation between RNA and protein levels across all the samples. **c** Pathways enriched among the genes with negative correlation between RNA and protein levels across all the samples.

A correlation between RNA and protein expression was performed on 34 cases (14 cases with +1q and 20 cases without +1q) with 6,731 genes that were common to both RNA and proteome datasets. Overall, a median RNA–protein correlation of 0.21 was observed for all genes across all the cases. In cases with +1q and without +1q, a similar global RNA-protein correlation profile was observed (Supplementary Fig. 1A). The overall RNA-protein correlation for all the genes was moderate (*r* = 0.429) between the two groups, with a similar correlation observed after excluding the genes located on 1q (*r* = 0.428) (Fig. 2A). Genes located on 1q exhibited a similar correlation (*r* = 0.454). Gene-set enrichment analysis was performed on genes with positive (Pearson correlation coefficient ≥0.6) and negative (Pearson correlation coefficient ≤ −0.1) RNA-protein correlations across all the samples. Genes with positive RNA-protein correlations were significantly enriched in focal adhesion, mitochondrial matrix function, negative regulation of apoptosis and metabolic processes (Fig. 2B). In contrast, genes showing negative RNA-protein correlations were significantly enriched in pathways related to protein processing in endoplasmic reticulum, protein transport and mRNA processing among others (Fig. 2C). We then examined the chromosomal location of these genes to assess if +1q influences RNA and protein expression. There was a higher number of genes from 1q among the positively correlated genes in cases with +1q compared to cases without +1q (Supplementary Fig. 1B). *SLAMF6*, *SLAMF1, CD48*, *FCGR3A*, and *UAP1*, located in 1q23, showed positive RNA-protein correlation in cases with and without +1q (Supplementary Fig. 1C). *SLAMF1*, *SLAMF6 and CD48* belong to signaling lymphocyte activation molecule (SLAM) family of receptors and are highly expressed on immune cells including B-cells. Owing to their high expression in MM, they are often exploited as potential targets for immunotherapeutic drugs^23,24^. *HAX1* and *PBXIP1* were among the top positively correlated genes in cases with +1q, and these genes showed no correlation in cases without +1q (Supplementary Fig. 1D, E).

### Differential expression analysis of +1q compared to non +1q groups identified overexpressed proteins belonging to 1q region

**Fig. 3:**
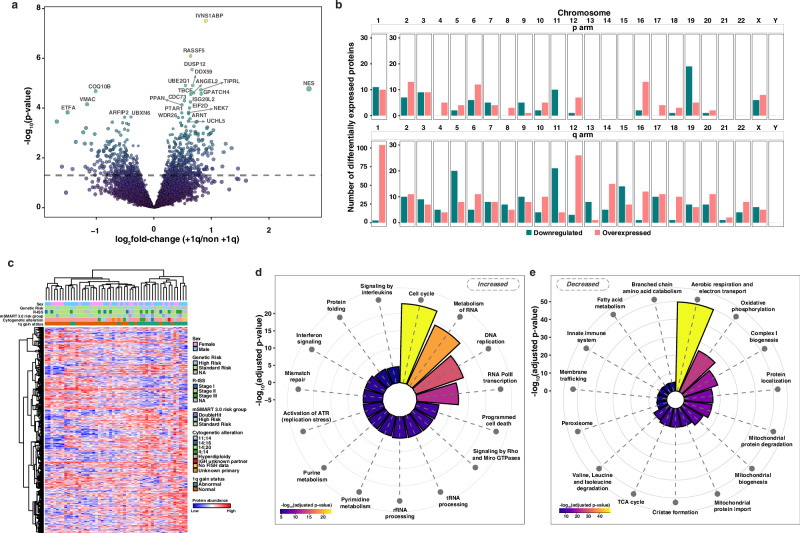
Differential expression analysis of proteins between cases with and without +1q. **a** Volcano plot showing the differential expression of proteins in cases with +1q compared to those without +1q (*p*-value ≤ 0.05). Top significantly differentially expressed proteins are labeled. **b** Distribution of the differentially expressed proteins between p and q arms across all the chromosomes. **c** Unsupervised hierarchical clustering of all the differentially expressed proteins. **d** Pathway enrichment analysis of the proteins that were significantly increased in cases with +1q compared to those without +1q. **e** Pathway enrichment analysis of the proteins that were significantly decreased in cases with +1q compared to those without +1q.

We next performed differential protein expression analysis to identify significantly altered proteins in cases with +1q compared to non +1q. Following correction for multiple hypothesis testing, 24 proteins met the adjusted significance threshold (adjusted *p*-value ≤ 0.05). Although MCL1 and ADAR, both previously associated with +1q, exhibited increased expression in the +1q group, they did not reach the adjusted significance threshold. Therefore, to comprehensively capture the proteomic alterations, we considered proteins with nominal p-value significance (*p*-value ≤ 0.05) that resulted in 404 proteins with increased expression and 256 proteins with decreased expression in cases with +1q (Fig. 3A, Supplementary Data 3). Mapping these differentially expressed proteins across all chromosomes showed a markedly higher number of proteins with increased expression from 1q chromosomal region (Fig. 3B). In total, 104 proteins showed increased expression in +1q cases with 60% of these proteins encoded by cytobands 1q21-1q25 (Supplementary Fig. 2A). These include well-known proteins previously described in multiple myeloma, such as NES, ETV3, UBE2Q1 and MCL1. Nestin, encoded by *NES* (1q23), was the top overexpressed protein identified in +1q cases. Its expression has previously been shown to be associated with different disease stages in MM^25^. Knockdown of ETV3 in myeloma cell lines decreased the proliferation ability and sensitized them to selinexor treatment^26^. Higher expression of UBE2Q1 in +1q cases was associated with poor overall survival and progression-free survival^13^. Notably, SLAMF1 (1q23), LAMTOR2 (1q22) and F11R (1q23) showed decreased expression in cases with +1q suggesting potential post-transcriptional regulation. Unsupervised clustering of the differentially expressed proteins is shown in Fig. 3C. These results indicate the potential gene dosage effect of +1q resulting in the increased expression of several proteins encoded by 1q region, which could play an important role in tumor behavior.

To further understand the cellular processes regulated by the differentially expressed proteins, we carried out pathway enrichment analysis. Cell cycle was the top significantly enriched pathway among the proteins with increased expression, followed by RNA metabolism, DNA replication and repair, programmed cell death, and protein folding. Cell cycle-related proteins with increased expression include CDK1, MCM complex, CHEK2, HAUS3, PSME3 and NEK7 (Supplementary Fig. 2B). Similarly, other proteins with increased expression include spliceosome components (PRPF3, PRPF4), RNA editing enzyme ADAR, and RNA exosome components (EXOSC10, EXOSC2, EXOSC1), which play an important role in RNA surveillance. Epigenetic regulators of programmed cell death such as DNMT1, EED, SUZ12 and RBBP7 were also increased in cases with +1q. Proteins with decreased expression were enriched in several metabolic pathways including aerobic respiration, oxidative phosphorylation, mitochondrial biogenesis and the TCA cycle (Fig. 3E). These proteins include several components from electron transport chain (NDUFV2, NDUFAF2, ATP5F1D, ATP5PF, COX16, COX17, COX19) and TCA cycle (IDH2, IDH3A, SUCLA2, ACAT1) (Supplementary Fig. 2C). Overall, these results emphasize the enrichment of the cell cycle pathway in cases with +1q and indicate potential therapeutic vulnerabilities that could be exploited for targeted treatment strategies.

### Protein network analysis highlighted the role of MYC targets in +1q MM

**Fig. 4:**
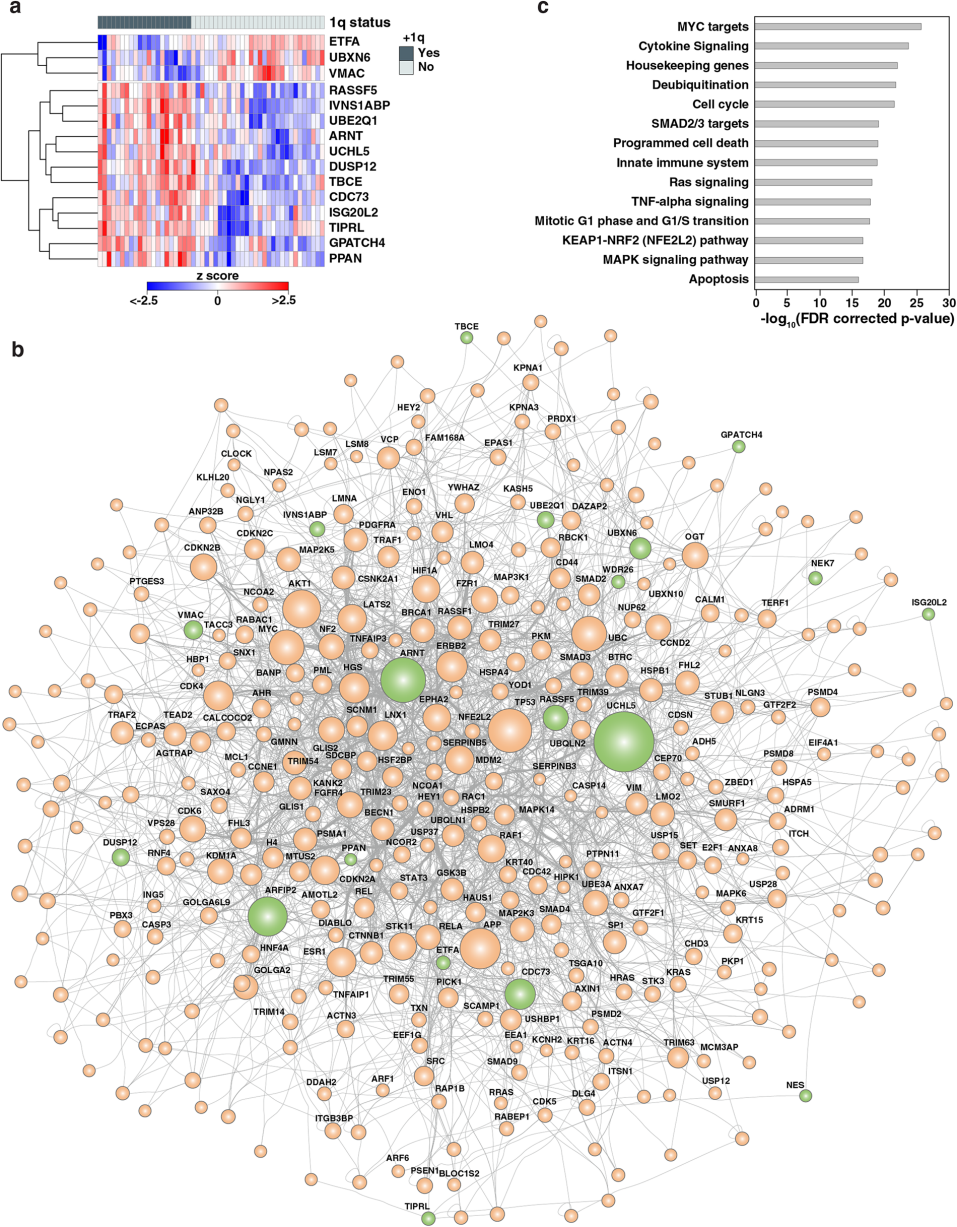
Protein-protein interaction network analysis of stringent differentially expressed proteins. **a** Heat map visualization of differentially expressed proteins that passed stringent FDR-corrected *p*-value cutoff of <0.05 and used as seeds for building the network. **b** Protein-protein interaction network of these seed proteins revealing major hub proteins. Green color nodes represent seed proteins used for building the network. Orange color nodes represent all the interacting partners of the seed proteins obtained from the BioGRID database **c** Pathway enrichment analysis of proteins represented in the protein-protein interaction network.

To understand the broader functional impact of proteins highly expressed in +1q MM, differentially expressed proteins with an adjusted p-value ≤ 0.05 were selected as seed proteins to build a protein-protein interaction network including their first-degree neighbors (Fig. 4A). This resulted in a large network, comprising 362 nodes (including the 15 filtered proteins) and 2390 edges indicating that these differentially expressed proteins are part of a tightly interconnected network, highlighting their functional coordination in cellular processes (Fig. 4B). This analysis identified UCHL5 as a major hub driver with a majority of edges connecting to other proteins. UCHL5 is a ubiquitin C-terminal hydrolase associated with the 26S proteasome and plays a crucial role in regulating the ubiquitylation levels of proteins destined for degradation. Increased expression of UCHL5 is known in multiple myeloma and drugs targeting UCHL5 have shown anti-tumor effects in myeloma cell lines^27,28^.

Pathway enrichment analysis performed on these proteins revealed significant enrichment of pathways known to be involved in MM (Fig. 4C). Interaction maps of enriched pathways illustrate the network of proteins involved in MYC transcriptional activation, cell cycle regulation, apoptosis, and the KEAP1-NRF2 (NFE2L2) pathway (Supplementary Fig. 3A–D). The enrichment of MYC targets underscores the critical role of MYC activation in MM, including cases with +1q, further highlighting its significance in disease progression. Consistent with these findings, a previous study reported an enrichment of *MYC* gene expression signature in +1q cases compared to cases without +1q^29^. Among these targets, differentially expressed proteins such as PPAN, TIPRL, DUSP12 and UBXN6, have not been well characterized in MM. Though the role of PPAN is not well established in cancer, studies showed that depletion of PPAN causes cell cycle arrest and induces apoptosis^30^. TOR signaling pathway regulator (TIPRL) is known to be an inhibitor of PP2A phosphatase activity regulating crucial biological processes such as apoptosis, cell proliferation and DNA damage response^31^. Overall, these results emphasize the potential role of MYC activation in +1q and its target candidates that need to be studied further.

### High expression of TIPRL is significantly associated with poor prognosis in MM

**Fig. 5:**
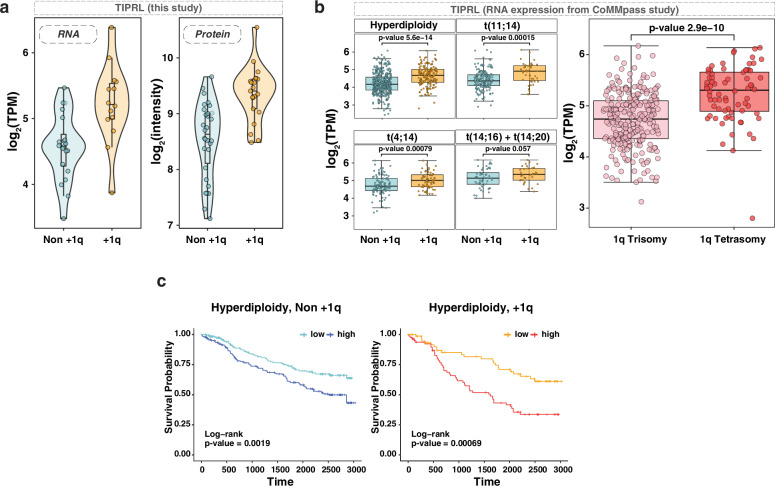
High expression of TIPRL is associated with poor prognostic performance. **a** RNA and protein expression of TIPRL in cases with +1q relative to cases without +1q. **b** RNA expression of TIPRL in CoMMpass cohort (left: +1q and non +1q cases across different cytogenetic alterations and right: expression in cases with 1q tetrasomy and 1q trisomy). **c** Kaplan–Meier plots showing overall survival in hyperdiploidy cases with and without +1q. All the cases were stratified based on the median expression of TIPRL in the CoMMpass cohort.

Among the stringent differentially expressed proteins encoded on chromosome 1q, TIPRL (log_2_fold-change 0.81, adjusted *p*-value 0.01) was one of the most significantly overexpressed proteins located on 1q24 and not well studied in MM. Increased expression of TIPRL was observed at both protein and RNA levels in cases with +1q (Fig. 5A). Its expression was higher in +1q across different genomic alterations (Supplementary Fig. 4A). Consistent with the proteomic analysis, we observed a statistically significant increase in expression of TIPRL in +1q cases in publicly available CoMMpass dataset. *TIPRL* transcript expression was consistently increased across different genomic alterations with +1q compared to without +1q, with notably higher expression in t(4;14) and t(14;16) groups (Fig. 5B). *TIPRL* showed higher expression in cases with 1q amplification compared to 1q gain. Next, we evaluated the prognostic value of TIPRL expression in MM using the publicly available CoMMpass dataset and observed that higher expression of TIPRL was associated with worse overall survival in hyperdiploidy MM irrespective of 1q status (Fig. 5C). No significant association was observed in t(4;14), t(11;14), t(14;16), and t(14;20) groups (Supplementary Fig. 4B–D). Overall, these results indicate that TIPRL is a novel differentially expressed protein with a potentially significant role in the pathogenesis of +1q MM.

## Discussion

MM is a highly heterogeneous disease with several genomic alterations shaping the molecular course of disease development^32^. Notably, t(11;14) translocation leads to an increased expression of cyclin D1 (*CCND1*), a protooncogene involved in cell cycle regulation, and it is associated with better disease prognosis compared to other high-risk abnormalities^33^. Similarly, t(4;14) translocation leads to increased expression of two oncogenes, *NSD2* and *FGFR3*, that drive tumor development and is considered as a high-risk abnormality with poor prognosis^34,35^. In contrast to these translocations, +1q has the potential to cause widespread dysregulation of multiple genes that are essential for cell proliferation and survival. Thus, identifying the genes affected by this alteration could offer critical insights into the biology of MM.

In this study, we conducted proteomic and transcriptomic analyses on enriched CD138+ plasma cells from MM cases with or without +1q to understand the effect of +1q. This study is particularly significant because, while previous research has largely focused on transcriptomic analyses^17,18^, proteomic investigations have been relatively limited largely because of the technical challenges associated with obtaining sufficient cell numbers for proteomic profiling after routine clinical testing. In this study, we addressed this limitation by performing proteomic analysis directly from protein pellets remaining after DNA and RNA extraction from CD138+ cells, thereby eliminating the need for additional sample material. This methodological advance not only enabled the present study but also provides a practical framework that can facilitate future proteomic investigations in multiple myeloma. Integrative analysis of RNA and protein expression showed several genes encoded by 1q region were positively correlated in +1q cases. These findings suggest that copy number gain in the 1q region manifests in increased RNA and protein expression and the potential dependence on these genes for tumor cell survival. Consistent with previous studies, differential expression analysis confirmed the increased expression of several well-known 1q-encoded proteins in cases with +1q, including MCL1^12^, NES^25^, ADAR^36^, ARNT^37^, UBE2Q1^13^. Many of these proteins play an important role in MM; e.g., MCL-1 is a key survival factor for plasma cells and its deletion results in a rapid loss of plasma cells in vivo^38^. Increased expression of ADAR1, an RNA editing protein, led to extensive hyperediting of transcriptome and enhancing oncogenic activity^36,39^. Additionally, we identified other proteins with increased expression located in 1q region that are not well studied such as DUSP12, PIP5K1A and TIPRL. DUSP12 is an atypical dual specificity phosphatase that is relatively understudied in cancer, however, its emerging role suggests that it promotes cell cycle progression by inhibiting apoptosis^40,41^. Increased expression of DUSP12 has been reported in diffuse large B-cell lymphoma^40^. Phosphatidylinositol-4-phosphate 5-kinase alpha (PIP5Kα) facilitates the synthesis of phosphatidylinositol 4,5-bisphosphate (PIP2), which subsequently activates the PI3K/AKT signaling pathway and is highly expressed in both breast^42^ and prostate cancer^43^. Despite these findings, 70% of the quantified proteins in the 1q region did not show significant differential expression indicating selective regulation of the proteins that are critical for cell survival. Further, we identified several non 1q-encoded genes that are differentially expressed in cases with +1q. Notably, decreased expression of BAD, a pro-apoptotic protein, indicates survival advantage of plasma cells in +1q myeloma. Kynurenine formamidase (AFMID), increased in +1q cases, is involved in tryptophan degradation pathway by catalyzing the conversion of N-formyl-L-kynurenine to L-kynurenine. Its expression was increased in colon cancer and it has been shown that MYC induced AFMID expression leading to increased kynurenine levels that were critical for maintaining cell viability^44^. Although the role of AFMID has not been studied in MM, elevated levels of 3-hydroxykynurenine have been reported in MM compared to MGUS and these levels were positively correlated with the percentage of clonal plasma cells^45^. This suggests a potential MYC-driven effect of AFMID expression, which could offer therapeutic opportunities targeting MYC pathway.

Pathway enrichment analyses showed enrichment of cell cycle pathway among proteins with increased expression that is consistent with the previous findings^46^. In addition, enrichment of proteins with increased expression that are involved in RNA metabolism pathway suggests transcriptome remodeling in +1q cases. For example, ADAR, involved in A-to-I editing in RNA, expression leads to hyperediting of MM transcriptome and promotes tumorigenicity in MM^36^, DNMT1 is involved in DNA methylation regulating gene expression in cancers including MM^47,48^ and METTL1, involved in N7-methylguanosine (m^7^G) modification of RNA, was overexpressed in AML^49^. Finally, enrichment of TCA cycle and oxidative phosphorylation pathways among the proteins with decreased expression in cases with +1q indicate a reliance on anaerobic metabolism in cases with +1q^50^. The interactome analysis provided additional insights into the functional connectivity of dysregulated proteins in +1q MM. The identification of UCHL5 as a central hub protein suggests a role for ubiquitin-proteasome system dysregulation in MM^27,28^. Notably, MYC targets were increased in cases with +1q reiterating the importance of MYC in +1q MM as previously described^29^. TIPRL, one of the novel proteins associated with +1q, was observed as one of such MYC targets. Its increased expression across different subtypes and in cases with 1q amplification compared to 1q gain strengthens the evidence that its expression is driven by +1q. High expression of TIPRL was associated with poor overall survival indicating its potential role in promoting tumor cell survival and proliferation. TIPRL is known to positively regulate mTOR pathway by inhibiting protein phosphatase 2A^31^ and its expression is elevated in different cancers including non-small cell lung cancer^45^, hepatocellular carcinoma^51^ and head and neck squamous cell carcinoma^52^. TIPRL also plays a role in inducing autophagy upon metabolic stress and knockdown of TIPRL led to increased apoptosis, autophagy inhibition and low tumor growth in a lung cancer xenograft model^53^. Given its role in TOR signaling and apoptosis regulation, further studies are warranted to determine the mechanistic role of TIPRL in multiple myeloma.

In conclusion, this study provides a proteomic characterization of molecular alterations associated with chromosome 1q gain in multiple myeloma. One of the major limitations of this study is the relatively small cohort size. Because +1q frequently co-occurs with other cytogenetic alterations, this study was not powered to analyze subgroups defined by +1q in combination with additional cytogenetic abnormalities, thereby limiting the ability to assess subgroup-specific differences. An additional limitation is the lack of high-resolution copy number data to define the genomic regions of gain across chromosome 1q. Future studies incorporating high-resolution genomic profiling will be required to refine gene dosage effects across the 1q arm.

Nevertheless, the identification of several well-known proteins dysregulated due to +1q supports the findings of this study. Additionally, the discovery of novel differentially expressed proteins points to potential therapeutic vulnerabilities that can be tailored to +1q MM. Further mechanistic studies are needed to reveal the role of these proteins in disease resistance mechanisms as +1q is associated with relapsed myeloma compared to newly diagnosed myeloma. These results also emphasize the significant role of genes encoded by 1q chromosomal region in MM, regardless of +1q status. For instance, elevated TIPRL expression was associated with poor overall survival in hyperdiploidy cases, independent of the +1q, suggesting its crucial role in MM.

## Methods

### Sample cohort

Bone marrow samples were collected after approval from the Institutional Review Board at Mayo Clinic, Rochester (IRBs 919-04 and 521-93), and with written informed consent from all participants in accordance with the Declaration of Helsinki. CD138+ plasma cells were enriched from bone marrow aspirates and processed using AllPrep DNA/RNA/Protein extraction kit (Qiagen) using manufacturer’s protocol for DNA and RNA extraction. The flow-through from the RNA extraction step was precipitated with advanced protein precipitation buffer (Qiagen) to obtain the protein fraction and the protein pellets were stored at -80˚C until further processing.

### RNA sequencing (RNA-seq)

RNA libraries were prepared using the TruSeq RNA sample prep kit v2 (Illumina, San Diego, CA) according to the manufacturer’s instructions. An internally developed RNA-seq analysis workflow (MAPRSeq v2.0.0) was used to perform read alignment, quality control, and expression quantification. Pair-ended reads were aligned by STAR against hg38 genome and transcriptome defined by ENSEMBL gene build 88, quality control across genes was performed with RSeQC (v2.3.2), gene counts were generated with featureCounts from Subread package (http://subread.sourceforge.net/, v1.4.4). For each sample, gene counts were normalized using Conditional Quantile Normalization method (CQN) and generate gene and exon expression levels scaled against gene lengths and sample read depths (RPKM, reads per kilo base-pair per million mapped reads).

### Correlation of RNA and protein expression

Pearson correlation between RNA and protein abundance of all genes was computed after segmenting the cohort into three groups: samples with gain-1q; samples with no gain-1q; and all samples. Similarly, Pearson correlation for genes located in chromosome 1q, and genes not located in chromosome 1q was computed. For results described in Fig. 2A, where the impact of gain-1q on RNA-protein correlation was assessed, Pearson correlation of the correlations was performed.

### Sample processing for quantitative proteomic analysis

The protein fractions extracted from CD138+ cells were subjected to overnight acetone precipitation with 5 volumes of chilled acetone at −20 °C. The resulting protein pellets were resuspended in 6 M urea in 50 mM TEABC buffer followed by reduction with 5 mM dithiothreitol for 45 minutes at room temperature and alkylation with 20 mM iodoacetamide for 45 min in the dark at room temperature. After adjusting the urea concentration to <1 M, proteins were digested overnight with LysC/trypsin at 37 °C. Subsequently, the tryptic peptides were acidified and cleaned using C_18_ stage tips on the AssayMAP Bravo liquid handling platform (Agilent Technologies, Santa Clara, USA).

### Spectral library construction

Peptides pooled from multiple samples were used to construct a spectral library. The pooled peptides were fractionated using basic pH reversed-phase high-performance liquid chromatography on an UltiMate 3000 HPLC system (Thermo Fisher Scientific, Waltham, MA). Briefly, the peptides were dissolved in solvent A (5 mM Ammonium formate, pH 9.0) and separated on a C_18_ XBridge column (1.7 µm, 150 ×1 mm, Waters) using a stepwise gradient of solvent B (5 mM Ammonium formate, pH 9.0, 90% acetonitrile). The absorbance of eluted peptides was measured at 280 nm. The total run time was 90 min and 24 fractions were collected using a concatenation strategy. Fractions were dried using a speed vac concentrator. Each fraction was analyzed in data dependent acquisition parallel accumulation-serial fragmentation (DDA-PASEF) mode on a timsTOF pro mass spectrometer (Bruker Daltonics, Bremen, Germany) coupled to UltiMate 3000 RSLCnano system. The peptides were initially loaded on a trap column (Halo C_18_ 2.7 µm EXP2 stem trap, Optimize Technologies) using solvent A (0.1% formic acid). Peptide separation was performed using a 3 to 28% gradient of solvent B (80% acetonitrile, 0.1% formic acid) on an analytical column (25 cm × 75 µm, 1.6 µm C_18_, IonOpticks) for a total run time of 120 min at a flow rate of 300 nL/min. In DDA-PASEF mode, precursor ions were analyzed in PASEF mode with an ion mobility range of 0.6–1.6 Vscm^−2^. TIMS accumulation and ramp times were both set to 100 ms resulting in a 100% duty cycle. MS/MS data were acquired with 10 PASEF scans per cycle. Isolation width was set to 2 m/z for precursor ions below 700 m/z and 3 m/z for those above 800 m/z. Collision energy was increased stepwise from 20 to 59 eV as a function of increasing ion mobility from 0.6 to 1.6 Vscm^−2^. Mass spectra were acquired over an m/z range of 100–1700. The spectral library was generated using Pulsar in Spectronaut 18 (version 18.1) with default parameters.

### Mass spectrometry data acquisition and analysis

Peptide samples were analyzed in diaPASEF mode on a timsTOF Pro mass spectrometer connected to an UltiMate 3000 RSLCnano system. The diaPASEF method was designed with variable isolation widths optimized on the precursor ion densities in the spectral library using py_diAID algorithm^54^. Each sample was spiked with indexed retention time standard peptides (Biognosys) before being subjected to diaPASEF analysis. Liquid chromatography separation was performed as described in the previous section on spectral library construction. diaPASEF scans were carried out with two ion mobility windows spanning 0.7–1.43 Vscm^−2^ and a mass range of 400–1 200 m/z. A total of 16 diaPASEF windows were analyzed per cycle, with a cycle time of 1.8 s. The DIA window sizes ranged from 15 m/z (smallest) to 100 m/z (largest). TIMS accumulation and ramp times were both set to 100 ms per scan, ensuring a 100% duty cycle. MS/MS fragmentation was performed using a linear ramp of collision energy from 20 to 59 eV for 1/K0 = 0.6 Vscm^−2^ to 20 eV at 1/K0 = 1.6 Vscm^−2^. Ion mobility and mass calibration were performed using three ESI Tuning Mix ions (m/z, 1/K0: 622.02, 0.98 Vscm^−2^, 922.01, 1.19 Vscm^−2^, 1221.99, and 1.38 Vscm^−2^).

Mass spectrometry data analysis for protein identification and quantitation was performed using Spectronaut software (version 18.1). The data were analyzed in a hybrid DIA mode using the default settings. Protein identification was performed using a combined protein database consisting of human UniProt database (20,501 entries) and an antibody variable region database. Trypsin/P was set as the cleavage enzyme, allowing a maximum of two missed cleavages. Carbamidomethylation of cysteines was set as a fixed modification, while protein N-terminal acetylation and oxidation of methionine were considered as variable modifications. A false discovery rate of 1% at protein-level was used to filter the protein identifications across the experiment. Precursor quantitation was performed based on peak area and cross-run normalization was enabled.

### Bioinformatics analysis

Statistical analysis was performed using the Perseus computational platform^55^. After the abundance values were log-transformed, proteins were included for further analysis if they were quantified in at least 60% of the samples in each group. Imputation was performed assuming a normal distribution of abundance using the default settings of width 0.3 and downshift 1.8 in Perseus. Differential expression analysis was performed using a Student’s 2-sample *t* test with a *p*-value cutoff of 0.05. Additional statistical filtering of proteins was performed using multiple hypothesis correction to identify high-confidence differentially expressed proteins. Protein-to-chromosome location mappings were downloaded from the UniProt database. Gene-set enrichment analysis was performed using the MSigDB database^56^. Gene sets with a FDR-adjusted *p*-value of 0.05 were considered significant and redundant gene sets were manually filtered. All data visualization was performed in R statistical environment (version 4.3.1) using in-house developed programs. Protein-protein interaction data was downloaded from the BioGRID database (release 4.4.222)^57^. Networks were visualized using Cytoscape software version 3.9.1 (https://www.cytoscape.org/) and sub-networks were extracted using the MCODE algorithm implemented within Cytoscape.

## Supplementary information

Supplementary_data_figures

Supplementary Data 1

Supplementary Data 2

Supplementary Data 3

## Data Availability

RNAseq data have been deposited to GEO under the accession number GSE315888. The mass spectrometry proteomics data have been deposited to the Proteome Xchange Consortium via the PRIDE partner repository with the dataset identifier PXD064132.
