# Supplementary material for: Proteomic profiling revealed unique disease biology associated with 1q abnormalities in multiple myeloma
Source: NPJ Precis Oncol. 2026 Apr 14;10:222. doi: 10.1038/s41698-026-01364-7 (PMC13269749; doi:10.1038/s41698-026-01364-7)

## SUPPLEMENTARY FIGURES

**Supplementary Figure 1: RNA-protein correlation of genes across cases with and without +1q. a.** Plot showing the RNA-protein Pearson correlation profile of genes across cases with and without +1q. **b.** Plot showing the RNA-protein Pearson correlation profile of genes on 1q (left) and genes on all chromosomes excluding on 1q region (right) across cases with and without +1q. **c.** RNA–protein correlation plots of SLAMF1 (left), SLAMF6 (middle), and CD48 (right) reveal positive correlations in both cases with and without +1q. **d.** Representative list of genes with top discordant correlation between cases with and without +1q. **e.** RNA-protein correlation plots of HAX1 (left) and PBXIP1 (right) indicating positive correlation only in cases with +1q.

**Supplementary Figure 2: Differential expression analysis of proteins between cases with and without +1q. a.** Cytoband-wise distribution of differentially expressed proteins encoded on chromosome 1q in cases with +1q compared with cases without +1q. **b.** Proteins with increased expression that were involved in cell cycle, metabolism of RNA and programmed cell death pathways. **c.** Proteins with decreased expression that were involved in aerobic respiration and electron transport, mitochondrial biogenesis and mitochondrial protein degradation.

**Supplementary Figure 3: Protein-protein interaction networks. a.** MYC targets **b.** Cell cycle **c.** Apoptosis **d.** KEAP1-NRF2 pathway.

**Supplementary Figure 4: Kaplan-Meier analysis of TIPRL expression in t(11;14) and t(14;16)+t(14;20) cases. a.** Protein expression of TIPRL in cases with and without +1q across different genomic alterations. **b.** Kaplan-Meier plots showing overall survival in t(4;14) cases with and without +1q. **c.** Kaplan-Meier plots showing overall survival in t(11;14) cases with and without +1q. **d.** Kaplan-Meier plots showing overall survival in t(14;16)+t(14;20) cases with and without

+1q. All the cases were stratified based on the median expression of TIPRL in the CoMMPass cohort.

## SUPPLEMENTARY DATA

**Supplementary Data 1:** Clinical and cytogenetic characteristics of the study cohort.

**Supplementary Data 2:** List of proteins identified in diaPASEF analysis of 51 MM samples.

**Supplementary Data 3:** List of differentially expressed proteins (p-value≤0.05) identified in cases with +1q compared to those without +1q.

# Supplementary Figure 1

**a** **b**

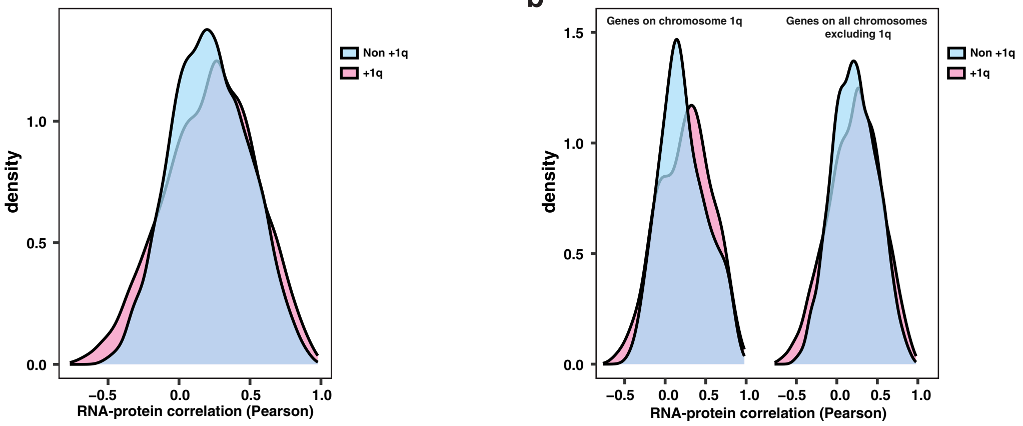


**c**

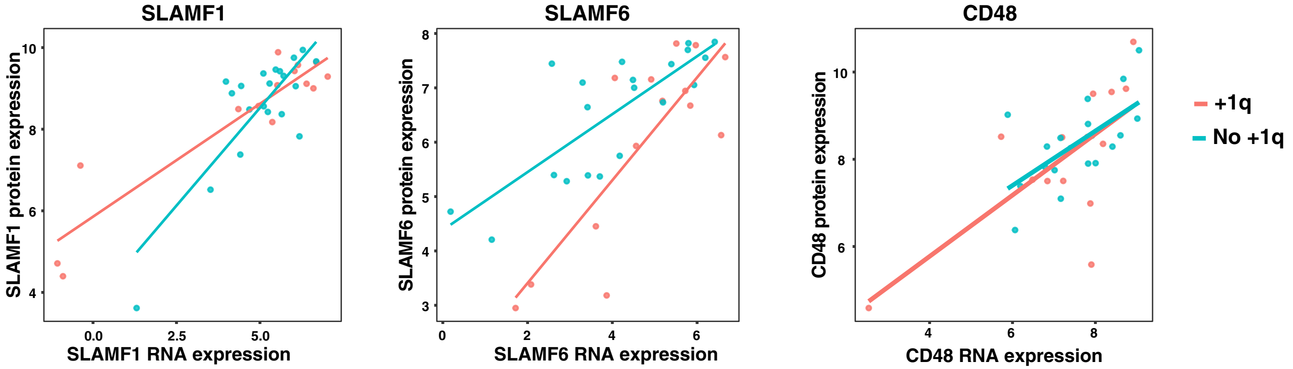


**d**

| Gene | RNA-protein correlation (samples with +1q) | RNA-protein correlation (samples without +1q) | Located in Chromosome 1q |
|---|---|---|---|
| *HAX1* | 0.7515391 | -0.04847302 | Yes |
| *KDM4A* | 0.7562403 | -0.0835936 | No |
| *GC* | 0.7719071 | -0.0144544 | No |
| *EPHX2* | 0.7194785 | -0.03065803 | No |
| *CC2D1A* | 0.7001536 | -0.05241669 | No |
| *EIF4E3* | 0.7889601 | -0.1412117 | No |
| *PBXIP1* | 0.8793934 | -0.0509151 | Yes |
| *C2orf88* | 0.75121 | -0.15251439 | No |
| *EIF2B3* | 0.7029153 | -0.21005958 | No |
| *TMLHE* | 0.7484476 | -0.01194797 | No |
| *DNAJB9* | 0.7185721 | -0.06923534 | No |
| *MMP9* | -0.100527596 | 0.7207968 | No |
| *TUBA4A* | -0.059931291 | 0.7228258 | No |
| *TFDP1* | -0.035361516 | 0.7455403 | No |
| *PUSL1* | -0.207867713 | 0.727367 | No |
| *SIRT6* | -0.413582627 | 0.7158317 | No |
| *C11orf54* | -0.006897794 | 0.7327291 | No |
| *SMOC1* | -0.042412489 | 0.7492597 | No |

**e**

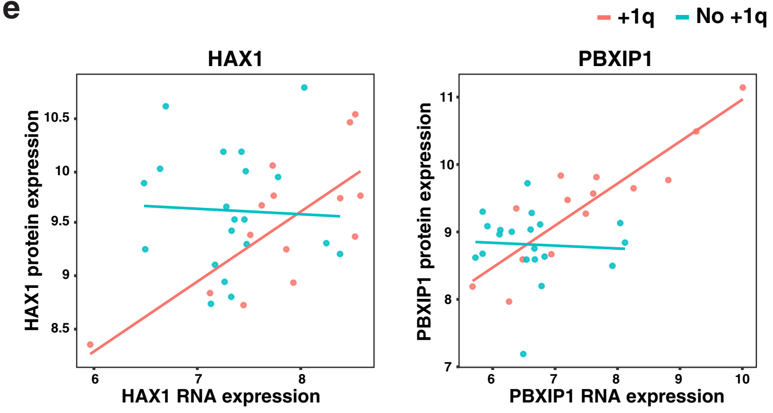

# Supplementary Figure 2

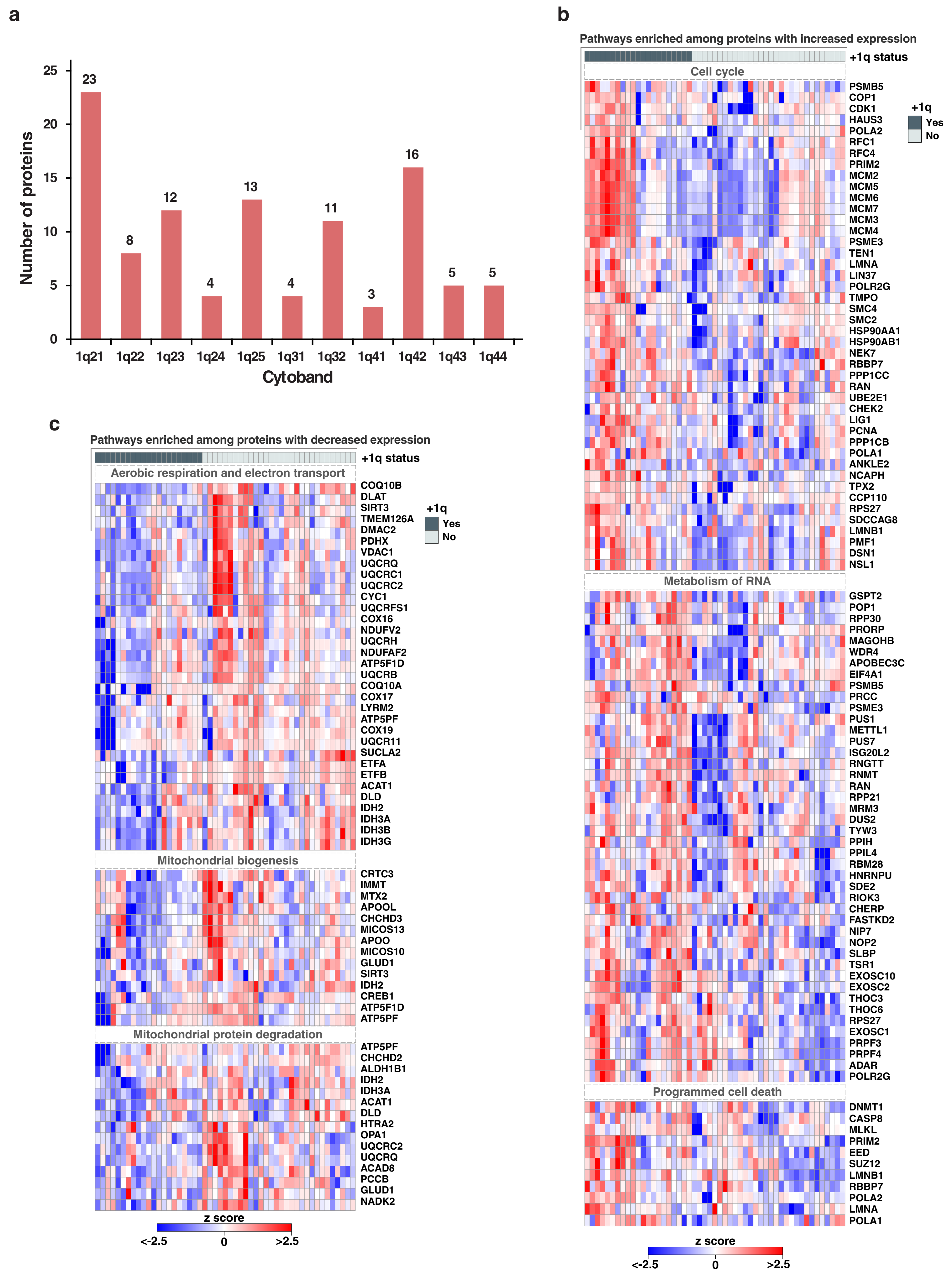

**Supplementary Figure 3**

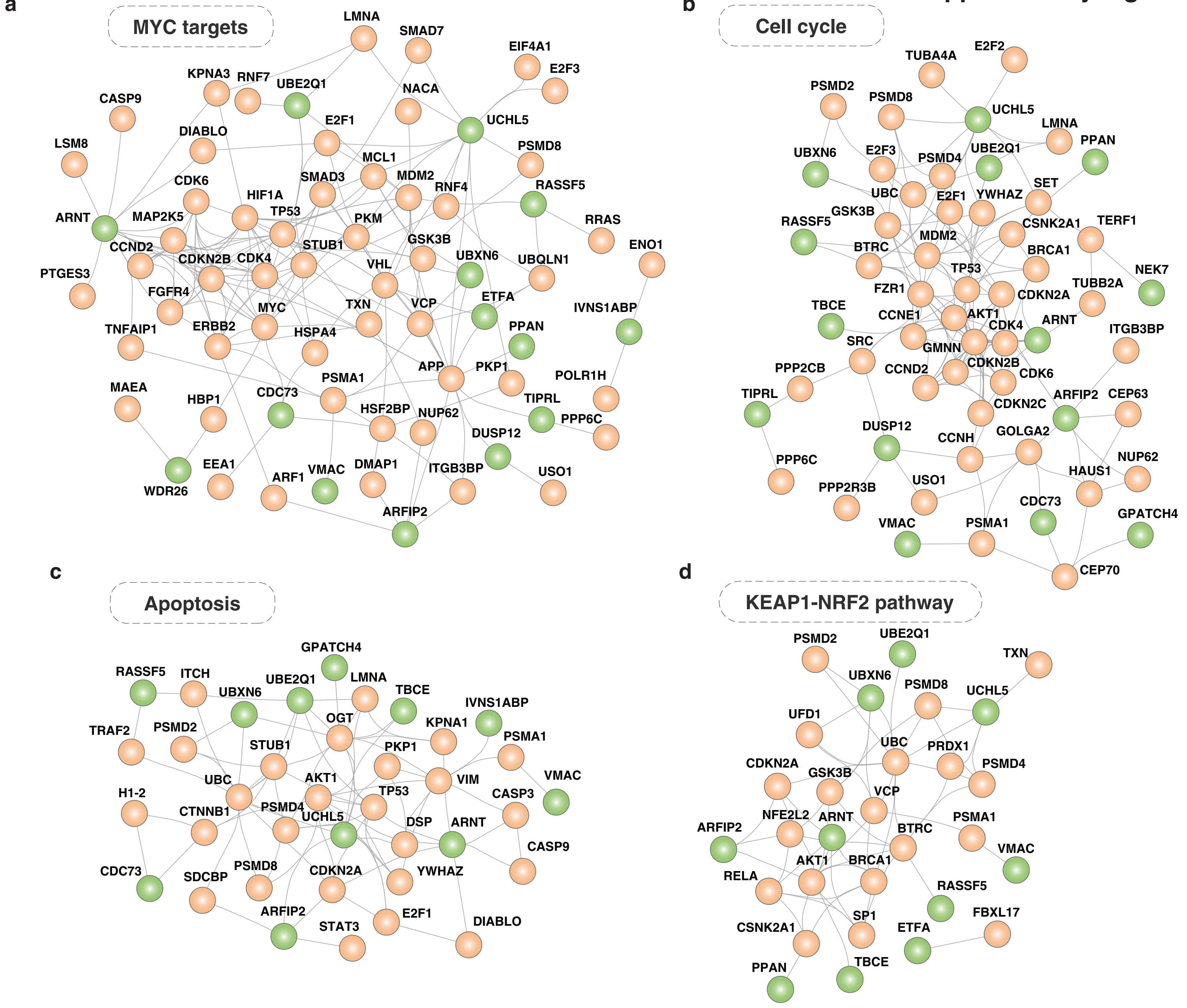

# Supplementary Figure 4

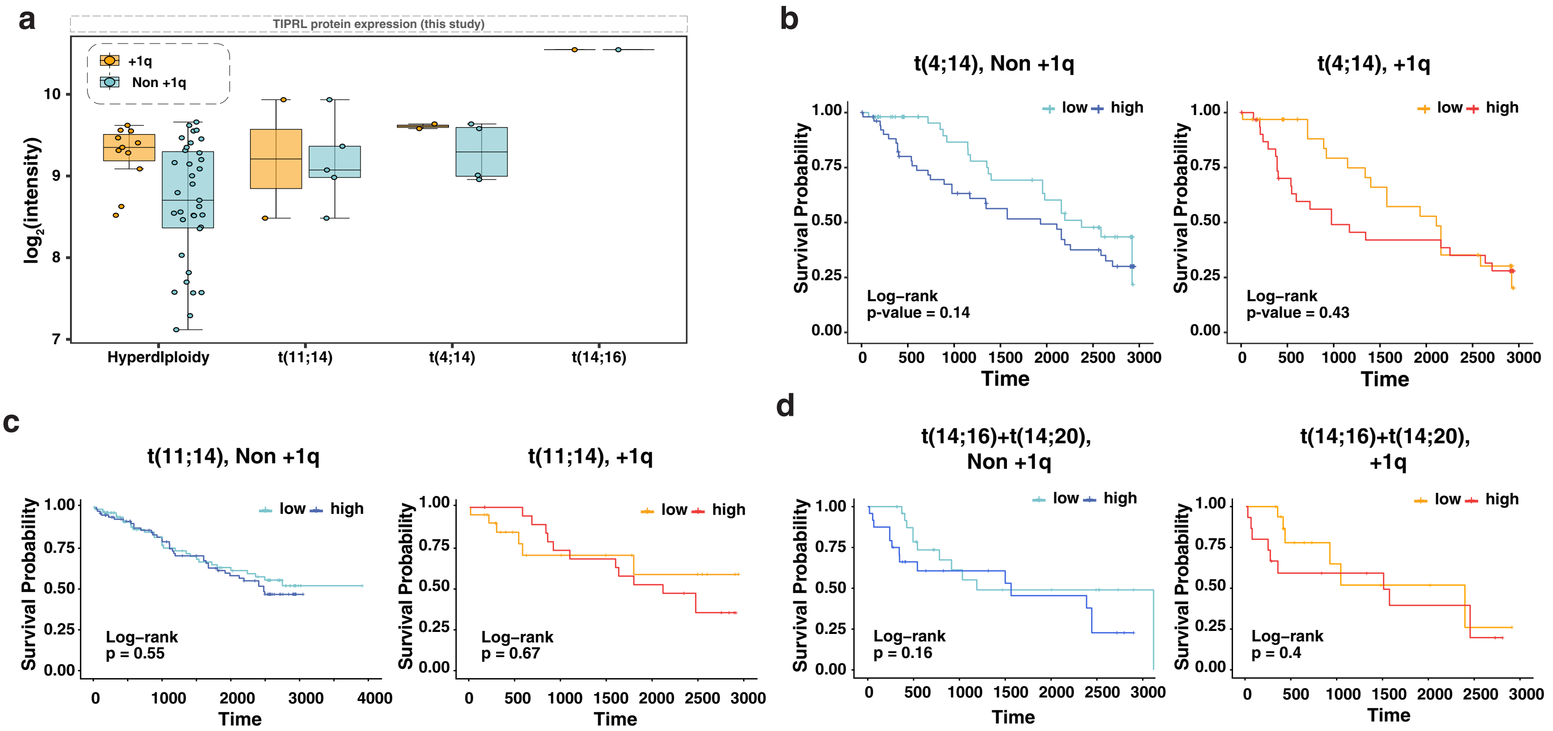

Supplement: Supplementary file 1 — Supplementary_data_figures [file 41698_2026_1364_MOESM1_ESM.pdf]
